# Comparative Analysis of Machine Learning Approaches for Fetal Movement Detection with Linear Acceleration and Angular Rate Signals

**DOI:** 10.3390/s25092944

**Published:** 2025-05-07

**Authors:** Lucy Spicher, Carrie Bell, Kathleen H. Sienko, Xun Huan

**Affiliations:** 1 Department of Mechanical Engineering, University of Michigan, Ann Arbor, MI 48109, USA; lspicher@umich.edu (L.S.); sienko@umich.edu (K.H.S.); 2 Department of Obstetrics and Gynecology, Michigan Medicine, Ann Arbor, MI 48109, USA; carriebe@med.umich.edu

**Keywords:** bi-directional long short-term memory (BiLSTM), convolutional neural network (CNN), fetal monitoring, inertial measurement units (IMUs), random forest (RF), spectrogram, time–frequency analysis, wearable sensors

## Abstract

Reduced fetal movement (RFM) can indicate that a fetus is at risk, but current monitoring methods provide only a “snapshot in time” of fetal health and require trained clinicians in clinical settings. To improve antenatal care, there is a need for continuous, objective fetal movement monitoring systems. Wearable sensors, like inertial measurement units (IMUs), offer a promising data-driven solution, but distinguishing fetal movements from maternal movements remains challenging. The potential benefits of using linear acceleration and angular rate data for fetal movement detection have not been fully explored. In this study, machine learning models were developed using linear acceleration and angular rate data from twenty-three participants who wore four abdominal IMUs and one chest reference while indicating perceived fetal movements with a handheld button. Random forest (RF), bi-directional long short-term memory (BiLSTM), and convolutional neural network (CNN) models were trained using hand-engineered features, time series data, and time–frequency spectrograms, respectively. The results showed that combining accelerometer and gyroscope data improved detection performance across all models compared to either one alone. CNN consistently outperformed other models but required larger datasets. RF and BiLSTM, while more sensitive to signal noise, offered reasonable performance with smaller datasets and greater interpretability.

## 1. Introduction

Prenatal care is essential for maternal and fetal health, with regular monitoring used to identify an at risk fetus and improve birth outcomes. Key indicators of fetal well being include fetal heart rate, movement, and muscle tone [1]. Among these indicators, maternal perception of fetal movement stands out as one of the oldest and most common methods of monitoring fetal health [2]. Reduced fetal movement (RFM) can signal complications: stillbirth, fetal growth restriction, congenital anomalies, and fetomaternal hemorrhage [3,4]. An estimated 25% of pregnancies in which mothers report RFM result in poor perinatal outcomes [3]. However, maternal perception inherently is subjective, influenced by factors such as activity level, placental position, and fetal sleep–wake cycles, and lacks standard guidelines for optimal number of movements [1,3,5,6,7]. Clinicians initiate antenatal testing where there is a report of RFM by completing a non-stress test. Fetal heart rate and uterine activity are monitored for 20 minutes for fetal heart rate accelerations or decelerations with or without contractions. Non-stress tests have significant false positive rates, resulting in additional testing to assess fetal wellbeing [1,8]. The contraction stress test provides information about fetal reactivity with uterine contractions and the biophysical profile measures fetal breathing, movement, tone, and amniotic fluid via ultrasound [1]. All these tests require trained clinicians and clinical settings and provide only a “snapshot in time” of fetal health, which may yield inconclusive results [1,8]. Despite the utility of clinical fetal monitoring, these methods can prompt cautious but sometimes unnecessary interventions—including early delivery, hospitalization, or more frequent monitoring [1,2,4,7,8]. Continuous, objective fetal movement monitoring could reduce reliance on subjective perception and isolated assessments, offering a more comprehensive understanding of fetal well being and improving antenatal care.

Wearable sensors offer a promising solution for continuous, objective fetal movement monitoring. However, distinguishing fetal movements from superimposed maternal movements in wearable sensor data continues to be a challenge [9,10,11,12]. Accelerometers have shown potential in controlled research environments when study participants are stationary. For instance, Khlif et al. achieved moderate accuracy (59%), sensitivity (76%), and specificity (55%) using a thresholding algorithm with three abdominal accelerometers [13]. Mesbah et al. applied signal preprocessing to remove maternal artifacts, achieving 95.8% accuracy but observing a sharp drop to 87.6% when maternal artifacts were included [9]. Delay et al. explored multiclass classification (e.g., fetal movement, maternal laughter, and respiratory motion), achieving an 86% true positive rate and 7% false positive rate with a single accelerometer [14]. Xu et al. achieved 86.6% accuracy and 84.2% F1 score using two accelerometers, but relied on synthetic minority oversampling [15]. These examples highlight the complexity of fetal movement detection, particularly the difficulty of distinguishing fetal movements from maternal motion—a challenge that becomes even greater in real-world settings outside controlled environments. Furthermore, differences in study populations and methodologies—such as gestational age, baseline fetal activity, preprocessing techniques, model architectures, and data representations—affect the results reported [16]. Comparing studies based on these metrics alone is not applicable to a broad patient population, as success reflects conditions and datasets specific to the study, and algorithms may not be generalizable. Understanding these nuances is crucial for interpreting research in this field and appreciating the variability inherent in fetal movement detection approaches.

Although evaluating the effectiveness of fetal movement detection approaches presents significant challenges, isolating and detecting fetal movements becomes particularly difficult when superimposed maternal movements are captured by wearable sensors [9,10,11,12]. In related biomedical applications where superimposed movements are also a concern (e.g., monitoring of sleep apnea in preterm infants, pulmonary conditions, and dietary behaviors), inertial measurement units (IMUs) have shown promise for accurate event detection [17,18,19,20]. Traditional accelerometer-based approaches detect fetal movement by identifying isolated changes in acceleration caused by abdominal deformation. When maternal movement occurs, a mixture of signals from both fetal and maternal motion are captured [9,10,11,12]. Angular rate gyroscopes, although rarely used to date in fetal movement detection, measure rotational motion and could help distinguish between the superimposed movements [21,22]. During maternal movement, the torso can be approximated as a rigid body, leading to similar gyroscope readings across sensors [23,24]. Thus, combining accelerometers with gyroscopes may detect localized abdominal deformation, potentially allowing for the identification of fetal movement in the presence of maternal motion.

Only one study to date has reported using both accelerometer and gyroscopic data obtained from IMUs for fetal movement detection [25]. The recent preprint by Alwis et al. demonstrated that a fusion deep learning model combining a convolutional neural network (CNN) and long short-term member (LSTM) layers achieved 88% accuracy, 0.86 sensitivity, and 0.91 specificity using spectrogram-based time–frequency representations [25]. While this study highlighted the potential of IMUs for fetal movement detection, it did not address the distinct contributions of acceleration versus angular rate data or evaluate their potential combined benefits relative to an accelerometer-only approach. Furthermore, Alwis et al. relied exclusively on spectrogram features and a specific fusion model architecture, leaving questions about how alternative data representations and machine learning models may perform. 

Given the variability across study methodologies, comparing how different data representations affect performance is crucial for advancing fetal movement detection approaches. For example, hand-engineered features capture domain-specific patterns, time series characterize temporal dependencies, and time–frequency spectrograms offer insights into frequency and time dynamics simultaneously [9,14,25]. Comparing these approaches provides a valuable understanding of the strengths and limitations of each method and helps standardize evaluations in the field.

This study explores fetal movement detection using acceleration and angular rate signals from wearable IMUs. The primary contributions of this work are as follows: (1) a comparative analysis of accelerometer and angular rate gyroscope performance in detecting fetal movement, (2) an assessment of their combined performance to enhance detection accuracy, and (3) an evaluation of various machine learning models trained on three distinct data representations—hand-engineered features, time series data, and time–frequency spectrogram representations. Additionally, this study examines the effect of decision thresholds for binary classification on model performance and the effect of training set size on model robustness. This analysis aims to provide a clearer understanding of each approach’s benefits and limitations, addressing gaps in current models and offering insights into how more efficient, deployable solutions can be developed for real-world use.

## 2. Materials and Methods

### 2.1. Participants

Twenty-three participants were recruited for a single data collection session at the University of Michigan. Participants were selected based on the following criteria: between 18 and 49 years old, currently carrying a singleton pregnancy, and between 24 and 32 weeks of gestational age. Appendix A provides additional demographic information about the participant population. Exclusion criteria included a diagnosis of gestational diabetes, hypertension, or any known fetal disease or physical abnormalities, to minimize confounding factors that could affect movement patterns. Written informed consent was obtained from all participants, and the study protocol was reviewed and approved by the University of Michigan Institutional Review Board (HUM00204999).

### 2.2. Sensor System

**Figure 1:**
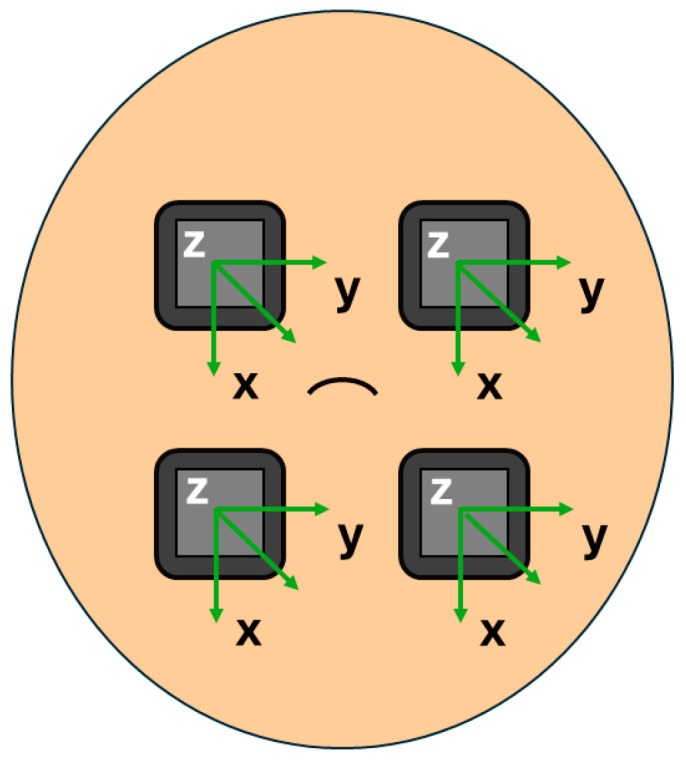
Position and alignment of four tri-axial IMUs on the maternal abdomen for fetal movement detection.

Data were collected using four tri-axial IMUs (Opal, APDM Inc., Portland, OR, USA) positioned around the participant’s umbilicus with a medical-grade adhesive. The x-axis of each IMU was aligned with gravity and the z-axis was perpendicular to the abdomen to ensure proper and consistent measurements of angular rates (Figure 1). To help distinguish between fetal and maternal movement, a chest IMU was added as a reference [9,13]. By approximating the torso as a rigid body, the angular rates measured by the chest and abdominal IMUs were assumed to be similar during maternal movement. Since the chest IMU was not expected to capture isolated abdominal deformations, it was considered useful for distinguishing maternal movement from fetal movement. The IMUs collected synchronized acceleration and angular rate data at a sampling rate of 128 Hz.

### 2.3. Experimental Protocol

At the start of each session, participants’ heights and weights were recorded, and the sensor system was fitted. Calibration data were collected by having participants perform a series of specified movements, which were later used to align the axes of the angular rate data. While accelerometer-only approaches have detected fetal movement in prior studies by capturing changes in acceleration tangential to the abdomen [10,13,14], approaches that use angular rate data have the potential to distinguish fetal movement from maternal motion but require an estimation of the sensors’ orientation [26]. Therefore, a Functional Alignment Method was employed to determine the anatomical axes relative to the IMU fixed frame of reference [26]. Since the IMU sensors were placed on the abdomen and the abdomen was assumed to move as a rigid body with rotation about the hip, the hip axis was used as a reference point to estimate IMU orientation. At the start of data collection, participants began from a standing position and were instructed to lean forward, bending at the hip, to a comfortable degree. This motion was repeated three times, and the calibration data from these hip-hinging movements were used to align the IMU axes (details related to signal processing are in Section 2.4).

Following calibration, participants were seated in a chair. Each participant held a unique IMU which functioned as a handheld toggle. When fetal movement was perceived, participants pressed the button on this IMU to mark the event as ground truth for subsequent movement detection analysis. Data collection was conducted in 10–15 min intervals to balance practical constraints with capturing meaningful fetal movement data. Since fetal movements often occur in clusters [27,28], shorter recording periods allowed for natural fluctuations while preventing participant fatigue and loss of focus, which could affect the accuracy of self-reported movements. Additionally, these intervals provided opportunities for participants to engage in behaviors that stimulate fetal movement, such as rubbing the abdomen or consuming a snack, increasing the likelihood of detecting activity [28]. While maternal perception captures only about 40% of true movements [6], simply increasing the recording duration would not necessarily yield more detected events. Instead, shorter trials helped sustain participant engagement, minimize fatigue-related artifacts, and effectively extend total observation time across the session. Each participant completed 2–3 trials, generating a total of 49 trials.

### 2.4. Signal Processing and Data Labeling

After data collection, the synchronized files from the IMUs were downloaded and reviewed for signal quality. Data from two participants were excluded due to signal acquisition issues. Raw acceleration and angular rate signals from each IMU were processed through a zero-phase bandpass filter (1–20 Hz) to reduce drift and noise [16].

For angular rate data, a Functional Alignment Method was applied to estimate each sensor’s orientation to improve differentiation between maternal and fetal movement signals [26]. During static standing, the global gravity vector was identified to establish a reference for the alignment of the vertical axis. Using rotation about the frontal axis, from the hip-hinging movements, the frontal and sagittal axes were identified. Each sensor’s x-axis was aligned with global gravity, the y-axis with the participant’s frontal axis, and the z-axis with the sagittal axis. This alignment allowed the angular rate data to capture deviations from expected rigid body motion—a key feature for distinguishing localized abdominal deformations caused by fetal movements from larger maternal movements. By aligning the axes this way, rotational changes inconsistent with whole-body movement patterns could be identified as potential fetal movements.

Axis alignment was not performed on acceleration data to preserve tangential fetal movement information on the z-axis, which is critical for capturing small, localized changes in abdominal acceleration that may be otherwise lost through axis realignment. Preserving the raw acceleration signal ensured that subtle changes in movement direction and magnitude—key indicators of fetal movement—were retained for analysis.

**Figure 2:**
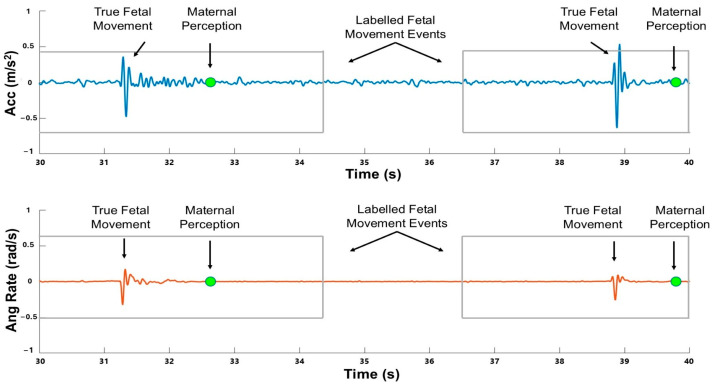
Example of fetal movement in the time series measurement data from accelerometer and gyroscope sensors. Bright green markers indicate maternal perception toggle presses, while labeled fetal movement events are defined to be within the gray boxes.

This combination of angular rate alignment and unaltered acceleration data leveraged each signal’s strengths: angular rate data for detecting deviations from maternal rigid body motion and acceleration data for capturing localized movement dynamics. An example of the time series measurements is provided in Figure 2.

Fetal movement events were defined 3.5 s prior to and 1.5 s after each recorded press of the handheld toggle to account for participant reaction time and fetal movement duration [29] (see Figure 2 for an example). The full time series data were then segmented by a window of fixed size. Binary labels were assigned to each window, where windows containing more than 10% overlap with event intervals were labeled as 1 and all others as 0. This 10% threshold, determined through visual inspection, effectively captured short fetal movements [30].

### 2.5. Model Training and Evaluation

From the full dataset, six trials were randomly selected for testing, six for validation, and the remaining thirty-seven for training. This division ensured that performance could be assessed on data unseen to the model, allowing for an evaluation of model generalization across different scenarios. After labeling, approximately 11% of windows were positively labeled. To mitigate data imbalance in the training set, two-thirds of negatively labeled windows were discarded to achieve an approximate 1:3 positive-to-negative ratio with 26% positive training windows [16]. Three dataset configurations were explored: (1) acceleration data only, (2) angular rate data only, and (3) a combination of both data types. For evaluation, the area under the receiver operating characteristic curve (AUROC), accuracy, sensitivity, specificity, positive predictive value (PPV), and F1 score were calculated to present a comprehensive view of model performance. Confusion matrices were also generated for each combination to provide a visual representation of classification performance.

To ensure robust and consistent evaluation, cross-validation was employed with a randomized repeated split approach [31]. Specifically, 10 sets of validation and testing splits were pre-selected prior to model training, with each split generated independently and without replacement to maintain diversity across trials. Model performance was then evaluated on the validation and testing sets to reflect its predictive capability on unseen data scenarios. This Monte Carlo cross-validation approach ensured that each model’s performance was averaged across 10 different splits, providing a robust estimate of generalization [31]. Furthermore, using the same validation and testing splits for all data representations ensured a fair and direct comparison of model performance across different dataset configurations. Other cross-validation approaches, such as k-fold, could also be employed. To assess the statistical significance of differences between models, *p*-values were calculated using paired statistical tests across the 10 validation and testing splits.

### 2.6. Feature-Based Approach: Random Forest Model

For the first data representation, all data trials were segmented into 0.5 s windows with no overlap. Statistical and correlation features were calculated for each axis and sensor, including the mean, standard deviation, and range. In addition, the cross-correlation between axes and the cross-correlation with the reference sensor for each axis were computed, as described in our previous study [30]. This process resulted in 75 acceleration features and 75 angular rate features per window. The features of each window were then used to train a random forest (RF) model. The model architecture consisted of an RF classifier with hyperparameters optimized via a random search based on the F1 score evaluated on the validation set. The final output was determined by aggregating the predictions of all trees using majority voting, based on a chosen threshold, to classify samples into {0, 1} [30].

### 2.7. Time Series Approach: BiLSTM Model

In contrast to the feature-based approach used for the first data representation, the second data representation used the time series data. Using information from the time domain maintains the natural temporal dynamics and memory of activity history, capturing transient accelerations and rotations that could indicate movement events. Preserving the time series structure may improve sensitivity to short, low-frequency bursts, characteristic of fetal movement [32]. 

For the second data representation, all data trials were again segmented into 0.5 s windows with no overlap. These windows were used directly as inputs into a machine learning model. The time series inputs were then used to train a bi-directional long short-term memory (BiLSTM) model, a type of recurrent neural network and variant to LSTM that is designed for capturing temporal dependencies from past and future contexts simultaneously [33]. Given that fetal movements often follow patterns or brief bursts, BiLSTM’s memory capability enables it to recognize and retain relevant time dependencies while ignoring noise or less relevant temporal artifacts.

**Figure 3:**
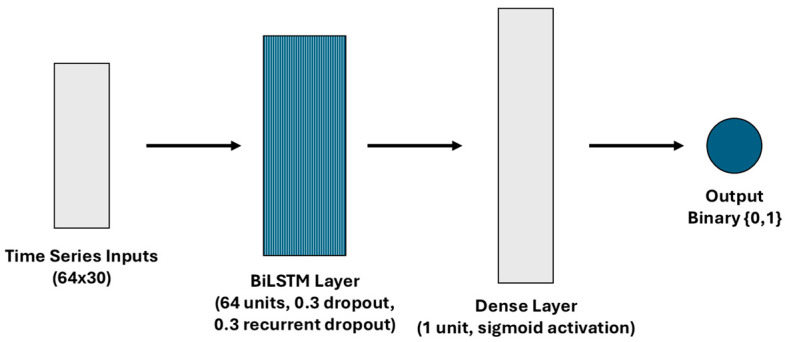
Model architecture for the time series approach.

The model architecture presented in Figure 3 included a single BiLSTM layer with 64 units regularized with 0.3 dropout and 0.3 recurrent dropout. The output from this layer was passed to a fully connected dense layer with sigmoid activation to produce a scalar value, which was rounded to {0,1} following a thresholding rule for binary classification. The model was trained using the Adam optimizer with binary cross-entropy loss.

### 2.8. Time–Frequency Approach: CNN Model

A time–frequency data representation was used for the third data representation to enable the model to leverage frequency-specific features, which may correspond to fetal movement rhythms or recurrent patterns that are not easily seen in the time domain [34]. Frequency–domain analysis has proven useful in other biomedical applications, particularly in distinguishing movement from background noise, and may improve model robustness against maternal motion artifacts [35]. Spectrograms also allow the model to focus on frequency components associated with fetal movement, improving sensitivity to movement events in noisy environments.

**Figure 4:**
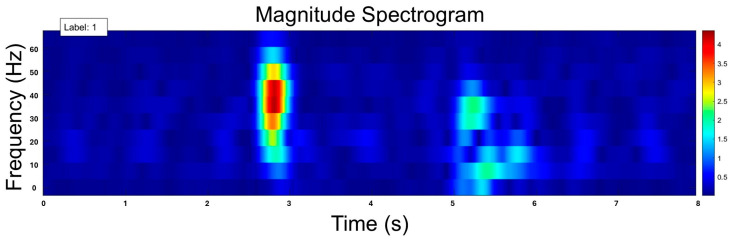
Example of fetal movement in the time–frequency spectrum.

For the third data representation, all data trials were segmented into overlapping 8 s windows with a 1 s stride. Although shorter windows (e.g., 0.5 s) are suitable for capturing fine-grained temporal patterns in feature-based or time series approaches [16], a longer window was necessary to balance the tradeoff between time and frequency resolution in the time–frequency representation [25]. Specifically, a 0.5 s window provided better time resolution, but poor frequency resolution, limiting the ability to capture meaningful frequency–domain patterns. Conversely, the 8 s window allowed for improved frequency resolution, which is crucial for distinguishing fetal movement patterns from maternal motion in the spectral domain. Within each 8 s window, the short-time Fourier transform (STFT) was computed using a sliding Hanning window of 16 samples with a stride length of 1 [25]. The magnitude of the STFT is known as the spectrogram time–frequency representation of the signal (Figure 4). A magnitude spectrogram was constructed for each window, and these spectrograms were used as inputs into a machine learning model.

Magnitude spectrograms were used to train a CNN. CNNs are effective for pattern recognition in image data, as they excel in identifying localized features and patterns [36]. By applying a CNN to frequency–domain representations, the model can learn frequency-specific movement characteristics while ignoring irrelevant noise, making it ideal for movement detection in an environment with variable maternal activity.

**Figure 5:**
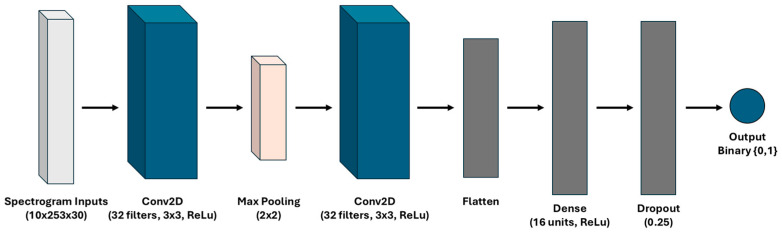
Model architecture for the time–frequency approach.

The model architecture, presented in Figure 5, consisted of an initial two-dimensional (2D) convolutional layer with 32 filters, each of size (3, 3), and ReLu activation. This layer was followed by a (2, 2) max-pooling layer to downsample the feature map. A second convolutional layer with 16 filters, each of size (3, 3), and ReLU activation was applied to further refine the extracted features. The output of the second convolutional layer was then flattened and passed to a dense layer with 16 units and ReLu activation, a dropout layer with rate 0.25, and a final output layer with sigmoid activation to produce a scalar value, which was rounded to {0, 1} following a thresholding rule for binary classification. The model was trained using the Adam optimizer and binary cross-entropy loss.

### 2.9. Thresholding Analysis and Training Set Size Reduction

In addition to the primary model evaluations, two exploratory analyses, thresholding and training set size reduction, were conducted to further understand model performance. Setting a good threshold for the machine learning models is an important part of creating an effective binary classification model. A standard 0.5 threshold is often not the best choice, and a smaller threshold (towards 0) tailors the model to predict more positives, while a larger threshold (towards 1) tailors the model to predict more negatives. This effect can also be quite sensitive at times, leading to fragile models. Therefore, a thresholding analysis was performed to vary the threshold from 0.1 to 0.9 in increments of 0.1.

Another consideration for data-driven modeling is whether the training set is sufficiently rich. This consideration is non-trivial and depends on the architecture and complexity of the models being used, the nonlinearity of the underlying data input–output relationship, and the settings and demographics being targeted. Nonetheless, sensitivity analysis can be performed by varying the training data size and providing insights for understanding model limitations and future data collection. The three models in this study were evaluated for robustness and sensitivity to data availability by training and testing them at 100%, 75%, 50%, and 25% of the full training set size.

## 3. Results

### 3.1. Summary of the Results

The results presented in this study demonstrate the comparative performance for detecting fetal movement when using only acceleration data, only angular rate data, and combining both. By evaluating three distinct data representations and models—hand-engineered features classified with RF, time series data analyzed with BiLSTM, and time–frequency spectrograms analyzed with CNN—this study highlights the strengths and limitations of each approach. Across all model types, the acceleration and angular rate datasets resulted in comparable performance. However, combining both types of sensor data consistently improved performance for all data representations. Additionally, model performance improved with increasing model complexity, specifically when transitioning from the simpler RF model to the more complex CNN.

### 3.2. Model Performance Across Sensor Types

**Figure 6:**
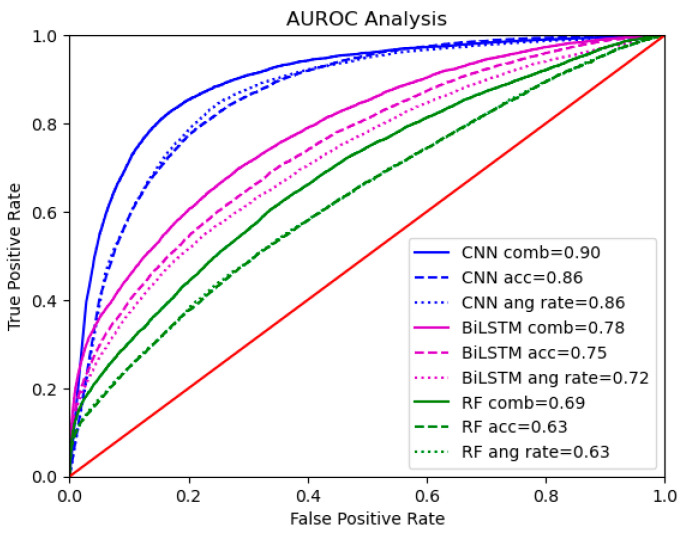
ROC curves and AUROC values (in legend) comparing acceleration, angular rate, and combined datasets with different models.

Mean receiver operating characteristic (ROC) curves, averaged across 10 testing splits, were generated for binary classification under varying thresholds (Figure 6), with the corresponding mean area under ROC (AUROC) values shown in the legend. The time–frequency spectrograms analyzed with CNN achieved the highest AUROC of 0.90 for the combined dataset, indicating “excellent” performance [37] in distinguishing fetal movement from non-movement. The single-mode acceleration and angular rate datasets achieved significantly lower AUROC scores of 0.86 (acceleration: *p*-value = 0.002; angular rate: *p*-value = 0.0002). Time series data trained with BiLSTM yielded “good” performance [37] with an AUROC of 0.78 for the combined dataset. The single-mode datasets scored 0.75 and 0.72, respectively—significantly lower than the combined dataset (acceleration: *p*-value = 0.0007; angular rate: *p*-value = 0.008). Hand-engineered features trained with RF achieved “sufficient” performance [37] with the lowest AUROC of 0.69 for the combined dataset. This lower value reflected the model’s limited capacity to capture the complex patterns of fetal movement through features localized to only the 0.5 s window. Single-mode datasets in this representation performed significantly lower than the combined dataset (acceleration: *p*-value = 4 × 10^−5^; angular rate: *p*-value = 0.00003) and were the lowest performing overall, with an AUROC of 0.63.

### 3.3. Model Performance Across Data Representations

**Figure 7:**
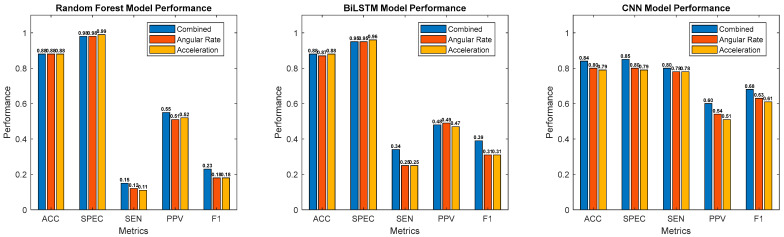
Performance metrics of RF, BiLSTM, and CNN models. Each histogram displays five metrics, accuracy (ACC), specificity (SPEC), sensitivity (SEN), positive predictive value (PPV), and F1 score, with bars indicating the model’s performance on combined, angular rate, and acceleration datasets.

**Figure 8:**
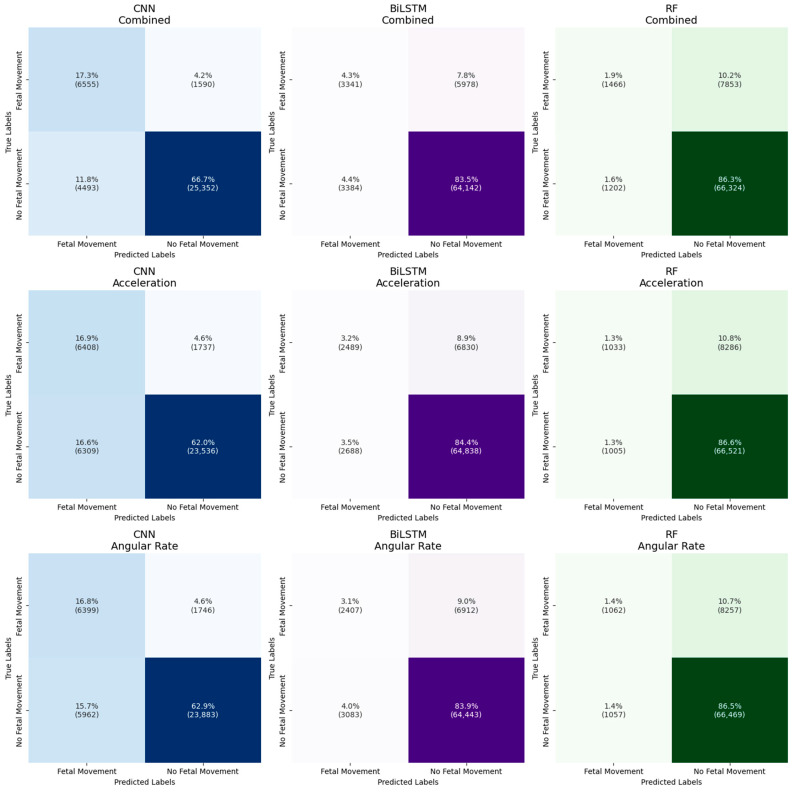
Confusion matrices showing the performance of the RF, BiLSTM, and CNN models across three datasets: acceleration, angular rate, and combined. The models’ classification performances are visualized in terms of true positives (**top left**), false positives (**bottom left**), false negatives (**top right**), and true negatives (**bottom right**). Each cell displays the percentage of the total number of samples, with the raw count shown in parentheses. RF matrices are shown in green, BiLSTM matrices in purple, and CNN matrices in blue. The combined datasets are positioned in the top row, acceleration in the second row, and angular rate in the third row. It is important to note that the models were evaluated using different window sizes: RF and BiLSTM used 0.5 s windows, while CNN used 8 s windows. This difference in window sizes may affect the distribution of predicted outcomes and should be considered when interpreting the results.

Accuracy, sensitivity, specificity, PPV, and F1 score, averaged over 10 testing splits and at a standard threshold of 0.5, are presented in Figure 7 for each dataset and model. Confusion matrices, presented in Figure 8, provide further insight into these results by showing the distribution of true positives, false positives, false negatives, and true negatives across models and datasets. To account for differing window sizes across models (e.g., CNN with 8 s windows vs. RF and BiLSTM with 0.5 s windows; see Section 2.8 for more details), the confusion matrices report percentages relative to the total number of samples, with raw counts included in parentheses. This presentation highlights both class imbalance and classifier behavior in a comparable format.

For the hand-engineered features classified with RF, all three datasets achieved similar accuracy, specificity, and PPV. For RF, sensitivity increased from 0.11 (acceleration: *p*-value = 0.005) and 0.12 (angular rate: *p*-value = 0.005) to 0.15 with the combined dataset. F1 score increased from 0.18 to 0.23 (acceleration: *p*-value = 0.002; angular rate: *p*-value = 0.001), and AUROC increased from 0.63 to 0.69 (acceleration: *p*-value = 4 × 10^−5^; angular rate: *p*-value = 3 × 10^−4^). Furthermore, the confusion matrices, shown in green, illustrate that the combined dataset exhibits more true positives (top left) and fewer false negatives (top right) compared to the single-mode datasets.

For the time series representations analyzed with BiLSTM, all three datasets achieved similar accuracy, specificity, and PPV. BiLSTM resulted in a sensitivity increase from 0.25 to 0.34 (acceleration: *p*-value = 0.0002, angular rate: *p*-value = 0.006), an F1 score increase from 0.31 to 0.39 (acceleration: *p*-value = 3 × 10^−5^; angular rate: *p*-value = 9 × 10^−4^), and an AUROC increase from 0.72 (acceleration: *p*-value = 0.0007) and 0.75 (angular rate: *p*-value = 0.008) to 0.78. The confusion matrices, shown in purple, further highlight this improvement by demonstrating the combined dataset’s ability to capture more true positives and fewer false negatives, which aligns with the higher sensitivity observed in the performance metrics. Across all datasets, the sensitivity (acceleration: *p*-value = 0.005; angular rate: *p*-value = 0.002; combined: *p*-value = 0.0008) and F1 score (acceleration: *p*-value = 0.002; angular rate: *p*-value = 0.0004; combined: *p*-value = 0.0007) of BiLSTM significantly outperformed RF.

Finally, for the time–frequency spectrograms analyzed with CNN, the combined dataset outperformed the single-mode datasets across all five performance metrics, with accuracy increasing from 0.79 (acceleration: *p*-value = 0.0006) and 0.80 (angular rate: *p*-value = 0.03) to 0.84, specificity increasing from 0.79 (acceleration: *p*-value = 0.02) and 0.80 (angular rate: *p*-value = 0.2) to 0.85, sensitivity increasing from 0.78 to 0.80 (acceleration: *p*-value = 0.7; angular rate: *p*-value = 0.7), PPV increasing from 0.51 (acceleration: *p*-value = 0.002) and 0.54 (angular rate: *p*-value = 0.005) to 0.60, F1 score from 0.61 (acceleration: *p*-value = 0.002) and 0.63 (angular rate: *p*-value = 0.001) to 0.68, and AUROC from 0.86 to 0.90 (acceleration: *p*-value = 0.002; angular rate: 0.0002). Although the combined dataset for the CNN model did not demonstrate statistically significant improvements in sensitivity over the single-mode datasets, CNN with the combined dataset achieved significantly higher sensitivity compared to RF (*p*-value = 1 × 10^−7^) and BiLSTM (*p*-value = 6 × 10^−7^) models trained on the same combined dataset, highlighting the superior capability of CNN in capturing true positive events. While maintaining accuracy and specificity similar to RF and BiLSTM, the CNN model achieved the highest performance in terms of sensitivity, PPV, and F1 score. Notably, the confusion matrices, shown in blue, further illustrate this increase in performance. The CNN model—particularly with the combined dataset—demonstrates a stronger balance between true positives and false negatives, and uniquely among all models and datasets, it also reduces the percentage of false positive predictions. 

### 3.4. Thresholding Analysis and Training Set Size Reduction

**Figure 9:**
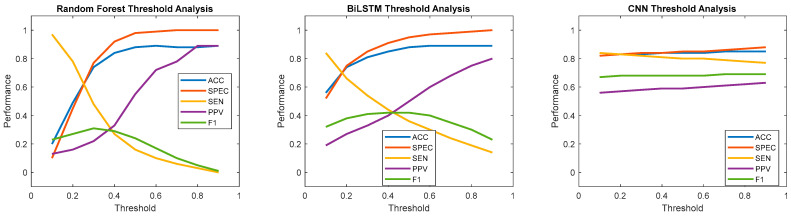
Performance of RF, BiLSTM, and CNN models across varying threshold values (0.1 to 0.9). Each line plot represents a different performance metric, illustrating how model performance changed with threshold adjustments.

Performance metrics resulting from varying the threshold for binary classification from 0.1 to 0.9 for each model type, trained and tested on the combined dataset, are reported in Figure 9. When evaluating RF across varying thresholds, accuracy and specificity increased from 0.1 to 0.5 and then stabilized at their best values. PPV also increased consistently, stabilizing only at a threshold of 0.8. Sensitivity continuously decreased as the threshold increased, and the F1 score increased slightly from 0.1 to 0.4 before decreasing thereafter. For BiLSTM, similar trends were observed but with higher minimum accuracy and specificity (just below 0.6 at a 0.1 threshold). Sensitivity ranged from 0.2 to 0.8 compared to 0 to 1 for RF. PPV and F1 scores were comparable but had less variation across thresholds. The performance metrics for CNN were notably stable across all thresholds. Like the first two approaches, accuracy and specificity increased as the threshold increased, while sensitivity decreased. The F1 score remained stable, while PPV showed a slight increase. This analysis highlights how threshold adjustments affected performance metrics differently across model types, with CNN demonstrating superior robustness compared to RF and BiLSTM.

**Figure 10:**
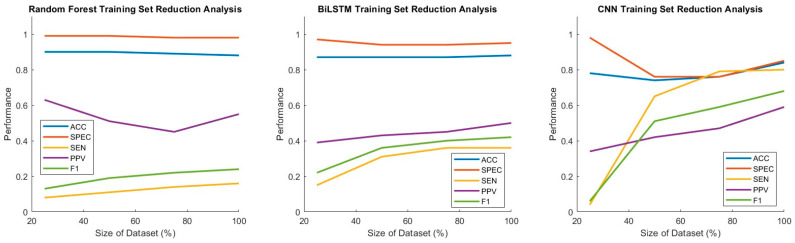
Performance of RF, BiLSTM, and CNN models across varying training set sizes (25–100%). Each line plot represents a different performance metric, highlighting how model performance changed with increasing training data.

**Figure 11:**
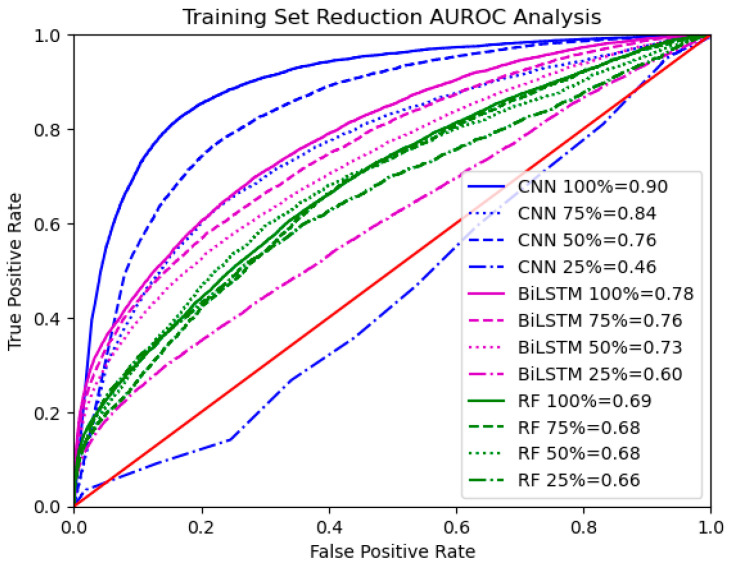
ROC curves and AUROC values (in legend) for all model types across varying training set sizes.

Performance metrics and ROC curves resulting from training and testing each model type on the combined dataset at 100%, 75%, 50%, and 25% of the training set size are reported in Figure 10 and Figure 11, respectively.

For RF, AUROC and other performance metrics remained low and relatively unchanged across different dataset sizes. For BiLSTM, AUROC, sensitivity, and F1 score improved steadily as training size increased. BiLSTM performed reasonably well, even at 50% of the dataset size, and continued to improve as more data were made available. For CNN, all performance metrics were poor at 25% but showed rapid improvement thereafter. CNN performed well at 50% and 75%, reaching its highest performance with 100% size.

## 4. Discussion

This study trained and tested machine learning models to detect fetal movement using acceleration and angular rate signals from wearable IMUs. Acceleration data, angular rate data, and combined datasets were used to train on hand-engineered features classified with RF, time series data analyzed with BiLSTM, and time–frequency spectrograms analyzed with CNN. Combining acceleration and angular rate data improved performance across all models. Time–frequency spectrograms analyzed with CNN yielded the best performance, followed by time series data analyzed with BiLSTM and hand-engineered features classified with RF, highlighting the benefits of more complex data representations and model architectures.

### 4.1. Model Performance Across Sensor Types

Single-mode sensors, whether capturing acceleration or angular rate data, performed similarly across all model types but were insufficient in fully capturing fetal movement patterns. However, combining acceleration and angular rate data consistently improved performance across all models. Notably, this combination improved sensitivity, F1 score, and AUROC.

Across all data types and models, the increased performance of the combined dataset suggested that the complementary information resulting from the combination of acceleration and angular rate datasets had discriminative power over either dataset on its own. The improvements in sensitivity, F1 score, and AUROC observed with the combined dataset were driven by an increase in true positives and a decrease in false negatives across all model types. For CNN, this improvement was also associated with a reduction in false positive predictions when combining datasets, further contributing to the model’s significantly higher PPV compared to single-mode datasets. These improvements suggest that combining acceleration and angular rate data enhances the models’ ability to accurately identify fetal movement events, improving the reliability of these methods for clinical use. Reducing false positive outputs prevents false reassurance of fetal well being, while fewer false negatives can help avoid unnecessary medical interventions.

### 4.2. Model Performance Across Data Representations

Among the three different data representations, hand-engineered features classified with RF resulted in the lowest AUROC (0.69), indicating a limited capacity to capture the complexity of fetal movement patterns. This low sensitivity was further emphasized by a relatively low number of true positives and a higher number of false negatives. While RF demonstrated similar specificity across datasets, its limited sensitivity and reliance on hand-engineered features likely contributed to its inability to accurately distinguish fetal movement from other signals. Furthermore, training set size analysis revealed that RF showed low sensitivity to data quantity, reinforcing its limitations in discriminating complex patterns in fetal movement data.

Time series data analyzed with BiLSTM improved AUROC (0.78), highlighting the power of sequential data for detecting fetal movement and its potential for enhancing sensitivity and reliability in clinical settings. The increase in true positives and decrease in false negatives observed with BiLSTM reflected its ability to better identify fetal movement events compared to RF. However, the decrease in PPV indicated a higher number of false positive predictions, possibly due to motion artifacts and overfitting. Despite its higher discriminative power compared to RF, BiLSTM still faced challenges in differentiating fetal movement from other signals. Additionally, both RF and BiLSTM were highly dependent on the choice of threshold, which can be challenging for deployment in real-world settings where signal noise can vary. Training set size analysis revealed a clear benefit for BiLSTM when larger dataset sizes were used, emphasizing its ability to better capture temporal patterns in fetal movement data as the dataset expanded.

Time–frequency spectrograms analyzed with CNN achieved the highest AUROC (0.90), demonstrating the value of preserving both temporal and frequency-specific information. The confusion matrices for CNN showed the highest percentage of true positives and lowest percentage of false negatives across all datasets, reflecting their ability to achieve increased sensitivity and reliability. Additionally, CNN reduced false positive predictions compared to BiLSTM, contributing to its improved PPV and F1 scores. Performance remained stable over all thresholds, suggesting greater robustness to noise and better suitability for real-world applications. Importantly, CNN performance remained consistently high with training sets above 25% of the total data size, indicating that CNN models excel when sufficient data are available, leveraging their capacity to recognize complex patterns in fetal movement data.

These results emphasized the unique ability of CNN to extract complex spatial and temporal patterns from spectrograms, distinguishing fetal movement from environmental noise and demonstrating the significant benefits of incorporating both time- and frequency-dependent features. Overall, CNN affirmed the importance of time–frequency representations for improving sensitivity and reliability, while BiLSTM outperformed RF, suggesting that more complex models led to improved performance in this problem setting. These findings emphasized the advantages of using both acceleration and angular rate signals and advanced model architectures.

### 4.3. Implications for Fetal Movement Detection

When considering the deployment of a fetal movement detection system in real-world settings, the tradeoffs between interpretability and model performance must be considered [38,39,40]. While combining acceleration and angular rate signals from IMUs was shown to be consistently more effective than individual sensors across all model types, the choice of model depends on the specific application and data available. RF excels at simpler tasks, such as detecting easily identifiable fetal movements in low-risk pregnancies, and offers quick, interpretable results through its relatively simple binary decision trees, making it suitable for environments with limited data or when immediate explainable feedback is essential [41]. In contrast, CNN excels at complex tasks, such as detecting subtle or noisy fetal movements in real-world environments, but it is also more computationally demanding to train and deploy, which may limit its feasibility for real-time use in devices with constrained power or battery life [42].

BiLSTM and CNN generally require larger datasets to achieve good performance, which may present another challenge in certain fetal monitoring contexts, such as when targeting at-home settings or specific demographics where data may be scarce [42]. For smaller datasets or when fetal movements are more pronounced, RF or BiLSTM may provide a more practical solution. However, when large datasets are available, as might be the case in large hospitals or other clinical settings, CNN should be favored for their consistent, robust performance.

Furthermore, the balance between model accuracy and interpretability is critical in real-world deployments [38,41,42]. In clinical settings, more complex models, like CNN, can offer high accuracy, but may require additional analysis or tools for interpretability, which may hinder clinician trust and delay their adoption [39,40]. On the other hand, for consumer-level wearables, where power consumption, size, and ease of use are more important, RF or BiLSTM may be more practical [4,39,43]. In conclusion, further advancements in sensor fusion and model development hold promise for enhancing fetal movement monitoring and providing a more reliable solution for both clinical and consumer applications.

### 4.4. Limitations and Future Work

While this study provided valuable insights into data-driven fetal movement detection using wearable IMUs, there are several important limitations and areas for future research before implementation in clinical or real-world settings is possible. Although machine learning models demonstrated strong performance across various sensor types and data representations, the “ground truth” labels in the current study were based on maternal perception, which is subjective and has intrinsic human error [4,6]. Counting perceived fetal movements requires attention and varies with maternal activity and fetal sleep cycles, so consequently some fetal movement may not be perceived. Maternal anxiety can negatively affect maternal perception, resulting in patients seeking care for inadequate movement [4,44,45]. Validating these models for clinical use requires comparison with ultrasound, which provides a detailed view of fetal activity [4,46]. Future work should aim to conduct prospective studies where fetal movement predicted from models using wearable IMU data is compared directly with real-time ultrasound observations to assess its true accuracy and reliability.

Another important consideration is the gestational age of the participant population. Participants in this study were recruited between 24 and 32 weeks of gestational age, as mothers can typically perceive fetal movement by the 20th week and there is an increased risk of labor after 32 weeks [47]. While the frequency of fetal movement is highest between 28 and 34 weeks, the risk of stillbirth increases with gestational age being highest after 41 weeks [48]. Future studies should consider prioritizing the recruitment of participants between 28 and 32 weeks to capture a greater number of fetal movements.

For a fetal movement detection system to be effective in real-world settings, it must be robust enough to handle a wide range of maternal activities of daily living (ADLs) without sacrificing performance [43]. Current models were all trained in controlled environments, but real-world use presents additional challenges, such as increased noise from maternal body movement, varying sensor placements, inconsistent data quality, and the lack of reliable data labeling, whether through maternal perception or ultrasound [4]. Future research should focus on testing these models in more diverse settings, such as during physical activity, sleep, and other routine behaviors, to evaluate their performance and robustness under real-world conditions. In addition, the development of adaptive algorithms that can adjust for such variability could be a promising direction for improving system reliability in non-clinical environments.

## 5. Conclusions

In conclusion, this study demonstrated that combining acceleration and angular rate data improves fetal movement detection across all data representations and model types. Time–frequency spectrograms analyzed with CNN achieved the best overall performance, highlighting the importance of preserving both temporal and frequency-specific information. BiLSTM outperformed RF, indicating that more complex models led to improved performance in this problem setting. These findings highlight the advantages of using acceleration and angular rate signals and advanced model architectures. While these results emphasized the potential of advanced machine learning models, balancing model complexity with interpretability is essential for real-world applications. Future work should validate these models against clinical ultrasonography and assess their robustness during everyday activities, paving the way for wearable technology in antenatal care to improve maternal and fetal health outcomes.

## Data Availability

The datasets presented in this article may be available on request from the corresponding author. The data are not publicly available due to ongoing analysis.

## Appendix A

### Appendix A.1

This appendix contains demographic data applicable to the participant population in which IMU signals were acquired. S09 and S16 were excluded from the analysis due to signal acquisition issues reported in Section 2.4. Gestational age (GA) refers to the number of weeks that have passed since the first day of the participant’s last menstrual period. Only the week was reported, e.g., 32 weeks, and 5 days is recorded as 32 weeks. Parity refers to the number of times a woman has given birth, where mothers in their first pregnancy are nulliparous and those with previous pregnancies are parity ≥ 1. GA and parity were self-reported by participants. Body mass index (BMI) is calculated based on the height and weight of the participant taken on the day of participation. A pre-pregnancy BMI was not collected. A BMI ≥ 30 is classified as obese. BMI was calculated according to Equation (A1). GA, parity, and BMI can impact a participant’s ability to perceive fetal movement [4] and may impact the maternal perception labels applied to this analysis.

(A1) $$BMI=\frac{weight\;(kg)}{{height\;(m)}^{2}}$$

Fetal movement follows a diurnal pattern, with stronger and more frequent movements generally occurring later in the day [28,47,49]. As a result, the time of day may influence both a participant’s ability to perceive fetal movement and the frequency and intensity of movements that occur. To account for this potential effect, session timing was recorded and categorized as follows: “morning” sessions began between 7 and 10 a.m., “afternoon” sessions began between 2 and 4 p.m., and “evening” sessions began at or after 5 p.m.

**Table A1:** Demographic information for the participant population included in this study related to the ability to perceive fetal movement.

Participant Identifier	GA (in Weeks)	Parity	BMI	Time of Day of Data Collection
S01	32	Parity ≥ 1	37.2	Evening
S02	28	Nulliparous	38.7	Morning
S03	32	Nulliparous	31.7	Afternoon
S04	32	Parity ≥ 1	36.4	Evening
S05	29	Nulliparous	28.2	Morning
S06	31	Nulliparous	28.8	Afternoon
S07	26	Parity ≥ 1	36.3	Afternoon
S08	26	Nulliparous	25.7	Evening
S10	25	Parity ≥ 1	27.7	Morning
S11	31	Nulliparous	25.0	Evening
S12	28	Nulliparous	23.5	Evening
S13	24	Not reported	27.1	Evening
S14	26	Parity ≥ 1	35.0	Evening
S15	28	Nulliparous	25.0	Evening
S17	32	Nulliparous	39.8	Evening
S18	27	Not reported	30.3	Evening
S19	25	Parity ≥ 1	27.1	Evening
S20	25	Parity ≥ 1	30.5	Evening
S21	30	Parity ≥ 1	35.9	Evening
S22	24	Parity ≥ 1	24.8	Evening
S23	27	Nulliparous	25.8	Morning
